# Associations between level of services integration and nurses’ workplace well-being

**DOI:** 10.1186/s12912-014-0050-x

**Published:** 2014-12-31

**Authors:** Caroline Longpré, Carl-Ardy Dubois, Eric Tchouaket Nguemeleu

**Affiliations:** Department of Nursing, University of Montreal. Centre for Training and Expertise in Nursing Administration Research (FERASI), Université du Québec en Outaouais, St-Jérôme Campus, 5 Saint-Joseph Street, Room 3212, Saint-Jérôme, Québec, J7Z 0B7 Canada; University of Montreal, Montreal, Quebec Canada; Department of Nursing, Université du Québec en Outaouais, St-Jérôme, Quebec, Canada

**Keywords:** Workplace well-being, Care and services integration processes, Nursing practice, Organizational and professional changes, Perceptions of change

## Abstract

**Background:**

To respond better to population needs, in recent years Quebec has invested in improving the integration of services and care pathways. Nurses are on the front lines of these transformation processes, which require them to adopt new clinical practices. This updating of practices can be a source of both satisfaction and stress. The aim of this study was to gain a better understanding of the relationship between the transformation processes underlying services integration and nurses’ workplace well-being.

**Method:**

This study was based on a descriptive cross-sectional correlational design. The target population included all nurses working in four care pathways in a Quebec healthcare establishment: palliative oncology services, mental health services, autonomy support for the elderly, and chronic obstructive pulmonary disease. In all, 107 nurses took part in the study and completed a questionnaire sent to them. Hierarchical linear regression analyses were used to examine the relationship between level of integration, measured using the Development Model for Integrated Care; nurses’ perceptions of organizational change, measured on four dimensions (challenge, responsibility, threat, control); and nurses’ workplace well-being, measured on three dimensions (negative stress, positive stress, satisfaction), as defined by the Flexihealth model.

**Results:**

Nurses in the palliative oncology care pathway, which was at a more advanced level of integration, presented a lower negative stress level and a higher positive stress level than did nurses in other care pathways. Their mean satisfaction score was also higher. More advanced integration was associated with nurses’ feeling less threatened, as well as improved workplace well-being. The perception of threat appeared to be a significant mediating variable in the relationship between level of integration and well-being.

**Conclusion:**

The association observed between level of services integration and workplace well-being contributes to a better understanding of nurses’ experiences in such situations. These results provide new perspectives on interventions that could be implemented to remedy the potential negative consequences of these types of transformations.

## Background

To ensure better integration of care and services, Quebec’s Ministry of Health and Social Services (MSSS) undertook extensive structural and organizational reform, beginning in 2003, that involved implementing a new service organization model based on the development of local service networks (LSNs) [1,2]. At the heart of each LSN is a health and social services centre (HSSC), created by merging local community health centres (CLSCs) with residential and long-term care centres (CHSLDs) and, in most cases, a hospital (CH). To fulfill their responsibility of ensuring accessibility, continuity, and quality of services for the populations they serve, HSSCs are organized into service programs that group together services and activities to meet the needs of specific populations or groups of persons with a common health or social services problem [2].

Several studies have highlighted the benefits associated with an integrated service approach, both for patients and for health system efficiency [3-8]. However, few studies have explored the benefits of this approach for healthcare providers, or the relationship that might exist between the transformation processes underlying service integration efforts and providers’ workplace well-being. In this article we examine this relationship by exploring nurses’ perceptions of these processes.

On several levels, integration mechanisms show promise for addressing the many gaps in today’s healthcare system, such as service fragmentation, among other things. However, implementing them is a major challenge for organizations, and particularly for nurses, who are among those most affected by these initiatives. Nurses need to meet the new requirements associated with these transformations by adjusting and updating their professional practice and developing new competencies [5,9,10].

Some authors have suggested that a better-integrated healthcare system would be, for providers, a source of satisfaction, challenge, motivation, creativity, pride, and well-being [6,11]. Certain key integration mechanisms, such as interprofessional collaboration, working in networks or interdisciplinary teams [12-14], the development of new forms of relationships or interactions within or between organizations [1,15], and the introduction of new technologies or procedures better suited to the services provided [16], have been associated with new opportunities for nurses in terms of, for example, exercising clinical leadership and having greater professional autonomy [11]. At the same time, some analysts have pointed out that certain elements that are symptomatic of poor integration, such as the lack of formal planning of services, low involvement of care providers in decision-making, philosophical differences related to care provision, or lack of support for collaborative practice, can be sources of stress for professionals [17].

Other studies have highlighted secondary impacts that can also be associated with integration, particularly with regard to the challenges involved in its implementation. The organizational changes involved in implementing an integrated system can be a source of anxiety and negative stressors that affect workplace quality of life. Occupational stressors associated with changing roles and responsibilities, with new requirements to be met, or with role ambiguities have been identified as sources of uncertainty, instability, conflictual relationships, emotional exhaustion, and anxiety [17-22]. Over recent years, as Quebec’s healthcare system has been restructured and services have been integrated, several studies have observed an overall decrease in nurses’ job satisfaction [23-26], a rise in absenteeism, and increased psychological distress [21,27-29].

Thus, service integration processes can produce two types of emotional reactions: positive reactions associated with the perceived benefits and stimulating challenges inherent in these processes, and negative reactions associated with the destabilization, fears, and anxieties engendered by the same processes. The impacts on workers’ well-being can manifest as stress or dissatisfaction when the demands of the environment exceed their personal resources or when changes in tasks or required competencies involve major adaptations [30]. The negative reactions often seen at the start of a change process, when destabilization occurs, might persist, but they might also give way to more positive reactions or emotions as workers integrate new ways of working or begin to perceive benefits [30-33].

Despite the potential impacts on nurses’ work experience and well-being of the change processes involved in implementing an integrated approach, to our knowledge no study has systematically examined the relationship between integration efforts, nurses’ perceptions of the changes, and their workplace well-being. Our aim in this study was to analyze the relationship between the change processes underlying service integration projects and nurses’ workplace well-being, by exploring nurses’ perceptions of these processes. We explored three research questions:

1. What is the relationship between the level of care integration and nurses’ perceptions of that integration process?
2. What is the relationship between the level of care integration and nurses’ workplace well-being?

- 2a) Is a more advanced level of integration associated with a reduction in nurses’ negative stress on the job?
- 2b) Is a more advanced level of care integration associated with an increase in nurses’ positive stress on the job?
- 2c) Is a more advanced level of care integration associated with an increase in nurses’ job satisfaction?

3. Is the relationship between the level of care integration and nurses’ workplace well-being mediated by nurses’ perceptions of that process?

### The reference framework

**Figure 1:**
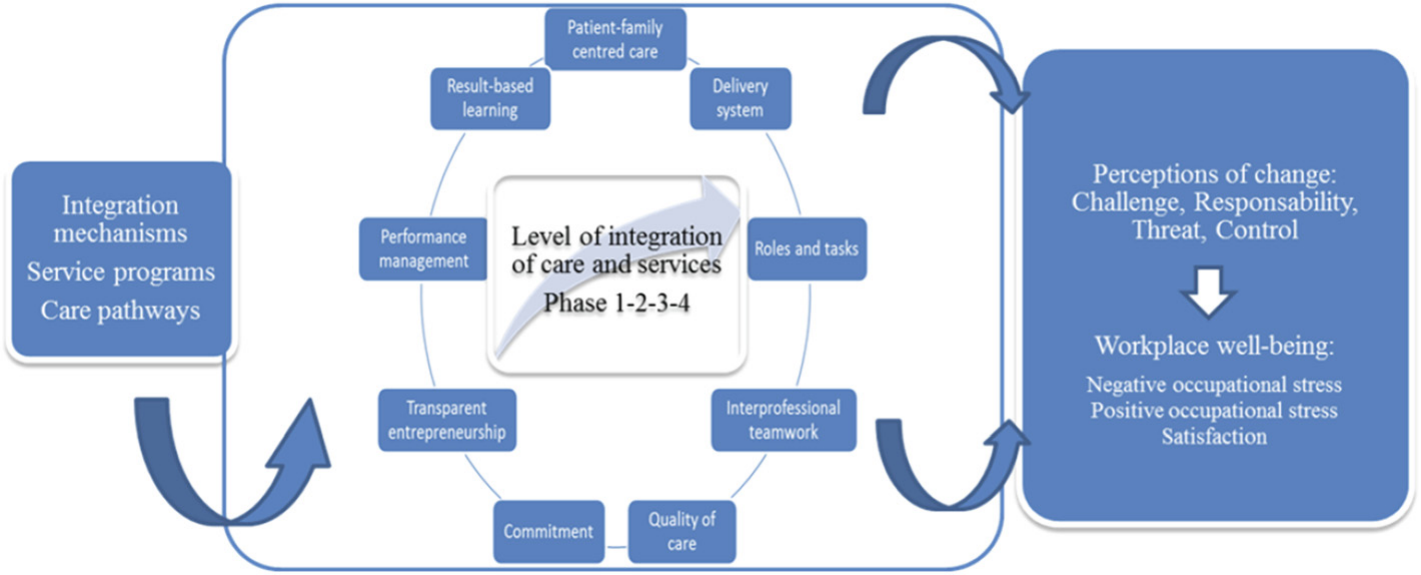
**Reference framework.** Inspired by the Flexihealth model of Vandenberghe et al. (2004) and the DMIC of Minkman et al. (2009, 2011).

The reference framework used for this study combines two models (Figure 1). The first, the Development Model for Integrated Care (DMIC) (translated, adapted, and validated) [34-36], describes the integration process by measuring 89 integrative activities grouped into nine practice dimensions. It is used to determine the level of advancement of a care integration process by positioning it in one of the four following phases: 1) initiative and design; 2) experimentation and execution; 3) expansion and monitoring; or 4) consolidation and transformation of the integration project.

The second framework, the Flexihealth model (adapted and validated) [37] was designed to analyze work situations that could potentially generate stress during change processes. Based on a transactional approach to professional stress and to its evaluation process [30], it links organizational change (the development of integration mechanisms) with the perceptual assessment of this change and workplace well-being.

The perceptual assessment of change was measured in terms of four dimensions: 1) *challenge*, which defines the extent to which the change is perceived as a challenge to overcome or an opportunity for development; 2) *responsibility*, which defines the extent to which the respondent perceives the organization to be responsible, or not, for the changes facing the workers; 3) *threat*, which defines the extent to which the change goes against the wishes of the respondent; and 4) *control*, which defines the extent to which the respondent feels empowered to modify the course of events.

Workplace well-being was operationalized using three constructs: negative stress, positive stress, and satisfaction. Negative stress, considered unhealthy, arises when individuals consider that the adaptation efforts required by their environment exceed their capacities and endanger their well-being [30], whereas positive stress represents a form of positive stimulation from the person’s work. Job satisfaction can manifest in various forms: overall, toward the organization, toward the job, or even just toward certain aspects of the job, such as the remuneration, one’s colleagues, etc. [37]. It is often considered to be the result of how the person assesses his job. A positive assessment or positive emotional reaction associated with the job will produce a certain level of job satisfaction, according to the person’s work experiences and expectations [38].

## Methods

### Research design

We used a descriptive cross-sectional correlational design [39] to analyze the relationships between level of integration and nurses’ workplace well-being, taking into account variables of perception.

### Study process

The study was conducted in an HSSC located in a semi-urban setting where the population was rising dramatically and becoming increasingly older. Since 2003, most HSSCs have been structured organizationally around service programs tailored to specific problems such as aging-related loss of autonomy, physical disabilities, intellectual disabilities and pervasive developmental disorders, youth in difficulty, addictions, mental health disorders, or physical health problems. Based on this organizational model, which had been implemented in the establishment under study, integrated care pathways were developed that were tailored to the growing needs of their clientele in a context of professional human resources shortages. As such, this HSSC presented a relevant laboratory for this study, as it was representative of a series of challenges facing HSSCs in Quebec as they strive to carry out their mandate to develop organizational models that will lead to better integrated care.

### Study setting

Of the five service programs at the HSSC, four agreed to take part in the study. In each program, we selected one care pathway based on the managers’ availability to participate in the study and the presence of a critical mass of potential respondents, with the aim of including pathways that were likely to be situated at different levels of advancement in their service integration process. The pathways we investigated were: chronic obstructive pulmonary disease (COPD), autonomy support for the elderly (ASE), palliative oncology services (POS), and mental health services (MHS).

### Population and sample

The target population consisted of all nursing personnel in all job categories (nursing assistants, nurses, nurse clinicians, counsellors, nurse navigators, liaison nurses, nurse practitioners, managers) working on the four targeted care pathways, except for orderlies, whose role is primarily one of support to nurses and who do not have professional status. The inclusion criteria were: being licensed to practice by their professional association (OIIQ – Ordre des infirmières et infirmiers du Québec or OIIAQ – Ordre des infirmières et infirmiers auxiliaires du Québec), working in one of the selected pathways, and having worked in that care pathway full- or part-time, day or night, for at least six months. To maximize the number of participants, all members of the nursing staff meeting these criteria were identified (n = 200: 35 in COPD, 70 in ASE, 35 in POS, and 35 in MHS) and contacted directly in the workplace, some individually and some in group meetings.

### Study variables and measurement instruments

We analyzed four types of variables: independent, mediating, dependent, and control.

#### Independent variable

The independent variable was the level of advancement of the integration process. This is a categorical variable with four possible values ranging from Phase 1 (least advanced) to Phase 4 (most advanced) [40] (Figure 1). A first part of this study consisted in determining the level of advancement of the integration process for each of the care pathways being investigated. Thus, the MHS and COPD pathways were at Phase 1 in their development, ASE at Phase 2, and POS at Phase 3. No pathway had reached Phase 4, the most advanced level.

#### Mediating variables

Echoing the work of Lazarus and Folkman [30], who showed that cognitive evaluation processes could act as mediators of stress response levels, this study took into account nurses’ perceptual assessment as a factor that can act as a mediator in the relationship between the integration process and workplace well-being. Specifically, perceptions of *challenge* (three items, α = 0.74), *responsibility* (three items, α = 0.78), *threat* (three items, α = 0.87), and *control* (two items, α not applicable), which altogether make up perceptual assessment, were measured using a five-point Likert-type scale ranging from *strongly disagree* to *strongly agree*.

#### Dependent variable

The dependent variable was nurses’ workplace well-being, The validated Positive and Negative Occupational Stress Inventory (PNOSI) consists of two parts: the negative stress scale, with nine items (α = 0.82, inter-item correlation 0.38), and the positive stress scale, with eight items (α = 0.88, inter-item correlation 0.39). All items were measured using a four-point Likert-type scales, ranging from 1 (*never or rarely*) to 4 (*always or nearly always*). The job satisfaction questionnaire consisted of two items (α = 0.69) measured using a five-point Likert-type scale ranging from *do not agree at all* to *agree completely*.

#### Control variables

The control variables refer to sociodemographic data on the respondents, including their function (clinician/manager), training (college/university), work shift (day/evening/rotation), and practice setting within the care pathway (CH, CLSC, CHSLD, family medicine group (FMG), ambulatory clinics, palliative care centre).

### Data collection process

All nursing staff meeting the inclusion criteria (n = 200) received a kit, which contained the study questionnaire, an information letter, an ethical considerations form approved by the research ethics committees of both the HSSC and the University of Montreal, and a stamped return envelope. The information letter explicitly stated that voluntary, anonymous return of the questionnaire constituted consent to participate in the research. To maximize response rate, reminders were provided two, three, and four weeks later, by telephone and directly within units.

### Analysis of results

First, we used descriptive statistics (mean, standard deviation) to draw up a profile of the respondents and determine average scores for perception and well-being variables [37]. As proposed by the Flexihealth model, a mean raw negative stress score between 15 and 23 corresponds to normal stress, while a score between 11 and 14 corresponds to a low level of negative stress, and a score of 24 and over, a high level. For positive stress, a mean raw score between 20 and 27 corresponds to a normal level, while a score below 20 corresponds to a low level of positive stress, and a score of 28 and over, a high level [37].

Second, we performed bi-variate and multicollinearity analyses to examine the correlations between level of integration and perceptions, level of integration and well-being, and the perception variables among themselves and in relation to the variables of perception and of well-being.

Third, we performed interaction analyses that examined the effect of combining the independent variable (level of integration for Phases 1 and 3, using Phase 2 as reference) and control variables (job function, training, work setting, shift) with each of the perception variables.

Fourth, we used hierarchical linear regression models to analyze the mediation effect of the perception variables in the relationship between level of integration and workplace well-being. More specifically, we examined three models: 1) the relationship between level of integration and nurses’ perceptions of the integration process; 2) the relationship between level of integration and well-being; and 3) the relationship between level of integration and well-being while taking into account all four perception variables simultaneously [41]. For each of the regressions, we studied multicollinearity using the tolerance coefficient and the variance inflation factor (VIF). The significance of the mediation effects was demonstrated using the Sobel test [42,43]. The analyses were performed using SPSS 20 and SAS 3.2 software at a 5% significance threshold.

## Results

### Profile of respondents

In all, 107 questionnaires (for a 54% response rate) were considered in this analysis. Respondents had, on average, worked 7.69 years (±5.08) at the HSSC and 7.75 years (±7.09) in their care pathway. Table 1 presents the detailed profile of respondents.

**Table 1:** **Profile of respondents**

**Variables**	**Dimensions of variables**	**Number of staff (n)**	**%**
Population/pathway	ASE	35	32.7
	MHS	28	26.2
	POS	24	22.4
	COPD	20	18.7
	Total	107	100
Function	Clinical^*^	85	79.4
	Management^**^	22	20.6
Training	College	34	31.8
	University	73	68.2
	Master’s	8	7.5
	Total	107	100
Work shift	Day	88	82.2
	Evening	18	16.8
	Rotation	1	0.9
	Total	107	100
Practice setting	CH	41	38.3
	CLSC	26	24.3
	CHSLD	20	18.7
	FMG	5	4.7
	Ambulatory clinic	13	12.2
	Palliative care centre	2	1.9
	Total	107	100

^*^nursing assistant, technician, clinician, counsellor, nurse navigator.

^**^manager, coordinator, director, assistant director.

### Descriptive results regarding nurses’ perceptions of the integration processes and nurses’ well-being, by care pathways

Compared to nurses in other pathways, the nurses working within the POS pathway reported above-average perceptions of *challenge*, *responsibility*, and *control*, and a below-average perception of *threat*. They had a weak level of negative stress, a high level of positive stress, and the highest satisfaction score among all pathways (Table 2).

**Table 2:** **Profile of variables by care pathways**

	**ASE**		**MHS**		**POS**		**COPD**		**Total**	
	**ẋ** ^*^	**σ** ^**^	**ẋ** ^*^	**σ** ^**^	**ẋ** ^*^	**σ** ^**^	**ẋ** ^*^	**σ** ^**^	**ẋ** ^*^	**σ** ^**^
Challenge	3.93	0.74	3.82	0.61	4.16	0.56	4.05	0.66	3.97	0.66
Responsibility	3.47	0.58	3.58	0.77	3.64	0.73	3.75	0.53	3.59	0.66
Threat	1.89	0.89	2.04	0.55	1.38	0.52	1.70	0.88	1.78	0.77
Control	3.60	1.01	3.68	0.94	3.77	1.02	3.25	1.21	3.59	1.03
Negative stress	15.89	3.79	16.00	3.39	13.04	2.66	14.25	2.59	14.97	3.43
Positive stress	23.92	4.35	24.14	3.92	27.69	3.38	26.45	3.32	25.30	4.12
Satisfaction	4.11	0.81	4.02	0.55	4.65	0.49	4.25	0.60	4.23	0.68

^*^ẋ = Mean.

^**^σ = Standard deviation.

### Analysis of relationships and interactions among study variables

Table 3 presents the matrix of correlations resulting from the bivariate analyses performed between level of integration, perception variables, and well-being variables. It can be seen that level of integration is significantly correlated with the well-being variables—positively with positive stress and satisfaction, negatively with negative stress, and significantly negatively with the perception of threat variable. Except for responsibility, all perception variables are correlated among themselves and with the well-being variables. With regard to the analysis of interactions, the effect of combining level of integration with control variables on each of the perception variables was not significant to 5% for most of the variables. For those that were significant, the analysis of multicollinearity with other variables in the model showed a VIF above 4 (6.112) and tolerance below 0.3 (0.164). Because of this, the interaction variables were not included in subsequent regressions.

**Table 3:** **Relationships between level of integration and variables of perception and well-being**

	**Challenge**	**Threat**	**Control**	**Responsibility**	**Satisfaction**	**Positive stress**	**Negative stress**	**Levels of integration**
Challen-ge								
Threat	r	−0.71						
	p	<0.001*						
	N	106						
Control	r	0.54	−0,57					
	P	<0.001*	<0.001*					
	N	105	105					
Respon-sability.	r	−0.08	−0.09	0.03				
	p	0.43	0.34	0.76				
	N	106	106	105				
Satisfa-ction	r	0.51	−0.44	0.39	0.12			
	p	<0.001*	<0.001*	<0.001*	0.21			
	N	106	106	105	106			
Stress(+)	r	0.56	−0.43	0.30	0.10	0.74		
	p	<0.001*	<0.001*	<0.001*	0.31	<0.001*		
	N	106	106	105	106	106		
Stress(−)	r	−0.47	0.44	−0.29	−0.02	−0.63	−0.67	
	p	<0.001*	<0.001*	<0.001*	0.85	<0.001*	<0.001*	
	N	106	106	105	106	106	107	
Level of integra-tion	r	0.15	−0.27	0.09	0.04	0.33	0.31	−0.30
	p	0.13	<0.001^*^	0.36	0.68	<0.001*	<0.001*	<0.001*
	N	106	106	105	106	106	107	107

^*^Significant p correlations.

r = Correlation coefficient.

p = Significiance.

### Mediating effect of perceptions between level of integration and well-beingModel 1: relationships between level of integration and nurses’ perceptual assessment of the process

After adjusting for the control variables (job function, training, shift, work setting), the results showed a statistically significant relationship between level of integration and two dimensions of perception: *threat* (p = 0.005) and *control* (p = 0.046) (Table 4). In Phase 3, the perception of *threat* is significantly lower and the perception of *control* is significantly higher than in Phase 2 (the reference phase). For both of these variables, differences between Phases 1 and 2 were non-significant.

**Table 4:** **Model 1: Relationship between level of integration and nurses’ perceptual assessment of that process**

	**Challenge**		**Threat**		**Control**		**Responsibility**	
	**β**	**p-value**	**β**	**p-value**	**β**	**p-value**	**β**	**p-value**
Constant	3.79^***^	<0.001	2.04^***^	<0.001	3.20^***^	<0.001	3.47^***^	<0.001
Function Ref.: NurseManager	0.56^**^	<0.01	−0.52^*^	<0.05	0.93^***^	<0.001	-	-
Phase Ref.: Phase 2								
Phase 1	0.14	0.243	−0.14	0.413	0.20	0.386	0.18	0.218
Phase 3	0.31	0.080	−0.60^**^	<0.01	0.57^*^	<0.05	0.17	0.349
Shift Evening	−0.44^**^	<0.01	0.31	0.107	-	-	-	-
∆R^2^	0.03	0.214	0.08^*^	<0.05	0.04	0.133	0.016	0.431

^*^p <0.05 ^**^p <0.01 ^***^p <0.001.

### Model 2: relationship between level of integration and nurses’ workplace well-being

The results showed statistically significant relationships between level of integration and negative stress (p = 0.002), positive stress (p = 0.001), and satisfaction (p = 0.011) (Table 5). In Phase 3, the level of negative stress was significantly lower and the levels of positive stress and satisfaction were significantly higher than in Phase 2. For these variables, there were no significant differences between Phases 1 and 2.

**Table 5:** **Model 2: Relationship between level of integration and well-being**

	**Satisfaction**		**Positive stress**		**Negative stress**	
	**β**	**p-value**	**β**	**p-value**	**β**	**p-value**
Constant	4.19^***^	<0.001	25.67^***^	<0.001	15.89^***^	<0.001
Training Ref.: College University	−^1^	-	−2.04^*^	<0.05	-	-
Phase Ref.: Phase 2						
Phase 1	−0.02	0.891	1.60	0.068	−0.62	0.403
Phase 3	0.46^*^	<0.05	3.61^**^	<0.01	−2.76^**^	<0.01
Shift Evening	−0.32	0.064	−3.04*	<0.05	-	-
∆R^2^	0.08^*^	<0.05	0.10^**^	<0.01	0.09**	<0.01

^*^p <0.05, ^**^p <0.01, ^***^p <0.001.

^1^Not included in the model.

### Model 3: simultaneous effect of level of integration and perceptions of *threat* and *control* on each of the well-being variables

- 3.a: Analysis of the mediating effect of the *threat* variable.
- When simultaneously applying level of integration and *threat* to each of the well-being variables one-by-one, the direct effect of level of integration on negative stress (p = 0.01) and on positive stress (p = 0.011) was significant, as was the effect of *threat* on negative stress (p <0.001) and positive stress (p <0.001). However, there is a slight attenuation of the effect of level of integration on both positive and negative stress in the presence of the mediating *threat* variable. Partial mediations are assumed. The direct effect of level of integration on *satisfaction* is not significant (p = 0.057), but the effect of *threat* on *satisfaction* is significant (p <0.001) (Table 6). Thus, there is a complete attenuation of the effect of level of integration, that is, a complete mediation. The results also show that as nurses’ perception of threat increases, their negative stress increases, their positive stress decreases, and they are less satisfied.
- 3.b. Analysis of the mediating effect of the *control* variable.
- When simultaneously applying level of integration and *control* to each of the well-being variables one-by-one, the direct effect of level of integration on negative stress (p <0.001), on positive stress (p = 0.003), and on satisfaction (p = 0.01) was significant, as was the effect of *control* on negative stress (p = 0.002), positive stress (p <0.001), and satisfaction (p <0.001) (Table 7). In the presence of the mediating *control* variable, there is a slight attenuation of the effect of level of integration on satisfaction and on positive stress, partial mediation is assumed, and there is a non-attenuated effect of level of integration on negative stress. For that reason, mediation is not assumed. Analyses also show that as nurses’ perception of control increases, their negative stress decreases, their positive stress increases, and they are more satisfied.

**Table 6:** **Model 3a: Simultaneous effect of level of integration and the threat variable on well-being variables**

	**Satisfaction**		**Positive stress**		**Negative stress**	
	**β**	**p-value**	**β**	**p-value**	**β**	**p-value**
Constant	4.77^***^	<0.001	29.06^***^	<0.001	12.73^***^	<0.001
Training Ref.: College University	-	-	−2.09^*^	<0.05	-	-
Phase Ref.: Phase 2						
Phase 1	−0.01	0.941	1.65*	<0.05	−0.63	0.353
Phase 3	0.33	0.057	2.56^*^	0.011	−2.17^*^	0.012
Shift Evening	−0.22	0.185	−2.79**	<0.01	-	-
Threat	−0.32***	<0.001	−1.82***	<0.001	1.67***	<0.001
∆R^2^	0.20^***^	<0.001	0.19^***^	<0.001	0.234***	<0.001

^*^p <0.05, ^**^p <0.01, ^***^p <0.001.

**Table 7:** **Model 3b: Simultaneous effect of level of integration and the control variable on well-being variables**

	**Satisfaction**		**Stress(+)**		**Stress(−)**	
	**β**	**p-value**	**β**	**p-value**	**β**	**p-value**
Constant	3.30^***^	<0.001	21.36^***^	<0.001	19.23^***^	<0.001
Training Ref: College University	-	-	−2.85^**^	<0.01	-	-
Phase Ref.: Phase 2						
Phase 1	0.01	0.950	1.96^**^	<0.01	−0.71	0.304
Phase 3	0.44^*^	<0.05	2.94^**^	<0.01	−3.08^***^	<0.001
Shift Evening	−0.26	0.107	−3.26**	<0.01	-	-
Control	0.24^***^	<0.001	1.33^***^	<0.001	−0.93^**^	<0.01
∆R^2^	0.22^***^	<0.001	0.18^***^	<0.001	0.206^***^	<0.001

^*^p <0.05, ^**^p <0.01, ^***^p <0.001.

**Figure 2:**
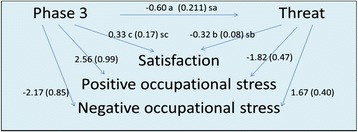
**Determinant relationships between level of integration, perception of threat, and nurses’ workplace well-being.** Controlling for all independent variables: a: non-standardized coefficient of the independent variable ‘Phase 3’ in the MHR** with threat Sa, Sb, Sc: standard error of the coefficient. b: non-standardized coefficient of the mediating variable ‘threat’ in the MHR** of the simultaneous effects of the independent variable and threat on satisfaction. c: non-standardized coefficient of the independent variable ‘Phase 3’ in the MHR** of the simultaneous effect of phase and threat on satisfaction. **MHR: multiple hierarchical regression.

The significance of the assumed mediation effects in the preceding analyses was confirmed using the Sobel test. The test demonstrated statistical significance for the indirect effect of level of integration on negative stress (p = 0.019), positive stress (p = 0.021), and *satisfaction* (p = 0.020), taking into account the mediation effect of *threat*. On the other hand, the mediation effect of *control* was not statistically significant (p >0.05). The hypothesis that *control* is a mediating variable was not confirmed. Lastly, perception of *threat* had a significant mediating effect in the relationship between level of integration and well-being. In summary, more advanced integration was associated with nurses’ feeling less threatened and with improved workplace well-being (Figure 2).

## Discussion

This study sheds new light on the mechanisms associated with the development of integrated care and services. While there is a great deal of literature examining service integration from the standpoint of service organization processes and their impacts on patients, less attention has been focused thus far on the impacts of such processes on health professionals, including nurses [8,44,45]. For example, satisfaction in relation to service integration has been copiously examined from the patients’ perspective, but very little from the care providers’ perspective, despite the fact that these changes are well known to generate workplace stress [46]. Thus, this study, based on the transactional theory of stress [30] embodied in the Flexihealth model, has shown how such processes, which fundamentally modify the work environment, influence the workplace well-being of care providers.

Analysis of the results reveals not only the direct impacts on nurses’ workplace well-being of the organizational or professional changes associated with these processes, but also the importance of the meaning nurses attribute to these changes and their experience [31]. By considering work situations as realities that are socially constructed by individuals based on their perceptions, the analysis approach we used highlights the interaction between individuals and their environment and recognizes their contribution to the implementation and continuation of transformation processes. This view echoes that of Terry and Callan (1997), cited by Vandenberghe et al., (2004) who emphasized the importance of taking into account both situational characteristics and individual perceptions, to understand how professionals adapt to changes in their organizations or in their practice [30]. Individuals will perceive their workplace as stressful or not depending on the meaning they attribute to the changes or events they experience [30].

This study revealed two principal types of reactions generated by integration processes, namely negative and positive reactions. Negative reactions, associated with the heavy transformation demands underlying integration, involved more negative stress, less positive stress, and less satisfaction; they were associated with the earliest and least advanced phase of the integration process, Phase 1. This phase, in which changes are initiated, is often marked by major disruptions. Positive reactions, on the other hand, involved greater positive stress, greater satisfaction, and less negative stress. They were associated with the most advanced level of integration, Phase 3, in which certain benefits associated with change start to become tangible. Such results are in line with the observations of Bareil, who explored the different stages of change processes, in which the actors involved go through several emotional and cognitive transitions as they come to adopt the change [31]. Resistances associated with shock (denial, a desire to continue working as usual) or reactions of fear and apathy that characterize the early phases of change are transformed in later phases into gradual engagement with the change and a shift toward feelings of pleasure and pride, related to perceived benefits or the acquisition of new competences. Another model is that of Lewin, which describes three stages of the change process, the first of which is “unfreezing”, or the period when habits and traditions are modified. It is followed by “transformation”, when new habits and competencies are formed, and culminates in “freezing”, the time in which new behaviours are internalized [47]. It might be hypothesized that the less positive results in terms of well-being observed in Phase 1 of the services integration process are linked to the considerable destabilization engendered by the development of these mechanisms or to the many emergent concerns associated with these changes [31]. In the more advanced phases, the positive results observed would be linked to greater mastery of the new ways of functioning and to the integration of new practices, which then become more natural and habitual, become aligned with other dimensions of daily practice, or are considered more satisfying [32,33].

With regard to the meaning attributed to change processes by nurses, this study offers two relevant contributions.

The first involves the relationships demonstrated between nurses’ perceptions and their well-being. The *control* and *challenge* variables were significantly positively associated with positive stress and satisfaction, and negatively associated with negative stress. Along the same lines, the Flexihealth study had previously associated perceptions of control and challenge related to change with a reduction in negative stress [37]. Many studies have shown the importance of control in contexts of change that generate increased resistance among workers to stressful events [37,48]. A meta-analysis of 88 studies showed that a perception of control was positively associated with desirable outcomes (professional satisfaction, job commitment, performance) and negatively associated with undesirable physiological or dysfunctional consequences (sleep disorders, emotional distress, absenteeism) [49,50]. Similar results were observed in the Flexihealth study, which showed that the perception of challenge had a significant effect on positive stress [37]. The change requires individuals to face and come to terms with numerous challenges. First they form a personal opinion about the relevance and quality of the proposed change, and then they must exert considerable effort to adopt and master the new competencies required [51].

The perception of threat was significantly positively associated with negative stress and negatively associated with satisfaction and positive stress. These results corroborate those of the Flexihealth study, which showed that the perception of threat elicited by organizational change could negatively affect workplace well-being, and that a minimal perception of threat had a significant effect on positive stress. When faced with change, individuals will assess the threat or challenge presented and their options for responding, recognizing that threat and challenge are, in fact, very closely related to each other. The individuals’ assessment of the perceived external demand (e.g. job requirements, number and scope of changes being imposed) and of their own resources or potential for adapting will determine the strength of the perceived threat and the consequent level of stress [37].

The Flexihealth study demonstrated a relationship between the perception of the organization’s responsibility and positive stress [37], in contrast to the results of this study, which found no association between responsibility and well-being variables. It might be that nurses give more weight to aspects of their work over which they can have control than to those that fall under the responsibility of the organization.

Taken together, these results associated with perception corroborate the conclusions of Mayssonnier, who pointed out the existence and importance of the relationship between perception and satisfaction (a component of well-being) by defining satisfaction as a fluctuating perception that evolves based on individuals’ needs and aspirations, and on the reality of their experience working in the organization [52-54].

The second contribution of this study relates to the determination of the role of perception variables as mediators between change and well-being. The perception variables of *control*, *challenge*, and *responsibility* were not correlated with level of integration and demonstrated no mediation effect between level of integration and well-being. On the other hand, the perception of threat was significantly negatively associated with level of integration. Moreover, the *threat* variable played a significant role of full mediation in the relationship between level of integration and well-being. The direct effect between level of integration and well-being was cancelled out by the introduction of the mediating variable (*threat*) into the model, which indicates the existence of a single dominant intermediary variable. Thus, in contrast to the other variables of perception, *threat* is an explanatory variable of this relationship. We might hypothesize that the absence of a mediation role in the other three variables could be due to the fact that those three variables are strongly correlated among themselves [37], and are thereby obscured by the *threat* variable. This phenomenon concurs with studies in the literature that suggest a care provider’s personal reaction to a situation perceived as threatening generates significant professional stress. Stress arises when the environment represents a threat for the individual, either because of excessive demands or because of unmet needs that hinder the individual’s work performance [55]. When confronted with a situation, individuals will seek to determine the extent to which it could affect their well-being [37,56,57]. In a context of organizational change, stress is seen as a dynamic process of assessment in which individuals see their environment as potentially threatening and likely to affect their well-being, and as something they do not feel they can contend with effectively. According to the Flexihealth model, this assessment is expressed in a variety of emotional reactions, with these variables being the mediating variables upon which the model is built and which could have an impact on their well-being and eventually even on their health [37]. The results of this study also contribute to enhancing this model.

### Study limitations and areas for future research

These results should be interpreted keeping in mind certain limitations. The first concerns sample size. With 107 respondents overall and fewer than 30 respondents in three of the four care pathways, it was not possible to carry out more in-depth analyses by pathway. A second limitation had to do with the number of sites. While the chosen study setting presented organizational and professional characteristics that were common to all HSSCs in Quebec, further studies would be needed to confirm these results and widen their scope to a diversity of contexts. It would be useful, in future studies, to use larger samples spread across more care settings. Larger-scale studies would allow for comparative analyses of the different settings, thereby strengthening the generalizability of the results. A third limitation is that, for reasons of feasibility, we did not take into account all the variables of the Flexihealth model [37], such as emotional reactions and personal assessment processes.

Applying the Flexihealth model fully in future studies would be useful for more in-depth analyses that would, for example, take into account individual variables (self-esteem, locus of control, social support) that might affect the evaluation process, or variables related to the impacts of organizational change on nurses’ physical and mental health.

## Conclusion

This study has contributed to establishing the relationship between nurses’ workplace well-being and level of integration, taking into account the mediating role of nurses’ perception of change. In the context of healthcare restructuring projects to develop more well-defined care pathways, our results reveal the potential impact of these changes on healthcare personnel. Three of the four care pathways studied were in the preliminary phases of their development, according to Minkman’s model (2011). These preliminary phases were associated with various perceptions of threats to nurses’ workplace well-being due to heightened negative stress. On the other hand, the nurses working in the palliative oncology services pathway, which was at a more advanced phase of integration, had a lower perception of threat and also presented a lower level of negative stress. Such results show the importance of paying careful attention to human resources management from the beginning of such projects when implementing change. Nurses should be given the necessary resources so they can exercise more control over events, both individually and collectively. Strategies to support workers, involve them in implementing change, and maintain and improve their health [31] should be developed to attenuate negative perceptions related to change, as well as negative consequences on workers’ health and well-being.

## Abbreviations

ASE: Autonomy support for the elderly
COPD: Chronic obstructive pulmonary disease
DMIC: Development model for integrated care
HSSC: Health and social services centre
CH: Hospital
CLSC: Local community health centre
LHN: Local health network
MHS: Mental health services
MSSS: Ministry of health and social services
POS: Palliative oncology services
CHSLD: Residential and long-term care facility
PNOSI: Positive and negative occupational stress inventory
